# Research on Surface Morphology of Gold Micro Bumps Based on Monte Carlo Method

**DOI:** 10.3390/mi14071345

**Published:** 2023-06-30

**Authors:** Haoyue Ji, Wenchao Tian, Hongwen Qian, Xiaodong Sun, Yongkun Wang, Lin Gu, Lihua Zheng

**Affiliations:** 1 The 58th Research Institute of China Electronics Technology Group Corporation, Wuxi 214062, China; edwardxxx5@163.com (H.Q.); sxd.8812@163.com (X.S.); gulin_110@163.com (L.G.); 1buer2@163.com (L.Z.); 2 School of Electro-Mechnical Engineering, Xidian University, Xi’an 710071, China; ykwang@xidian.edu.cn

**Keywords:** gold micro bumps, electrodeposition, molecular dynamics, Kinetic Monte Carlo, microscopic morphology, RMS roughness

## Abstract

In advanced packaging technology, the micro bump has become an important means of chip stacking and wafer interconnection. The reliability of micro bumps, which plays an important role in mechanical support, electrical connection, signal transmission and heat dissipation, determines the quality of chip packaging. Surface morphological defects are one of the main factors affecting the reliability of micro bumps, which are closely related to materials and bonding process parameters. In this paper, the electrodeposition process of preparing gold bumps is simulated at the atomic scale using the Kinetic Monte Carlo method. The differences in surface morphology and roughness of the plated layer are studied from a microscopic perspective under different deposition parameters. The results show that the gold micro bumps prepared by electrodeposition have better surface quality under conditions of lower deposition voltage, lower ion concentration and higher plating temperature, which can provide significant guidance for engineering applications.

## 1. Introduction

**Figure 1:**
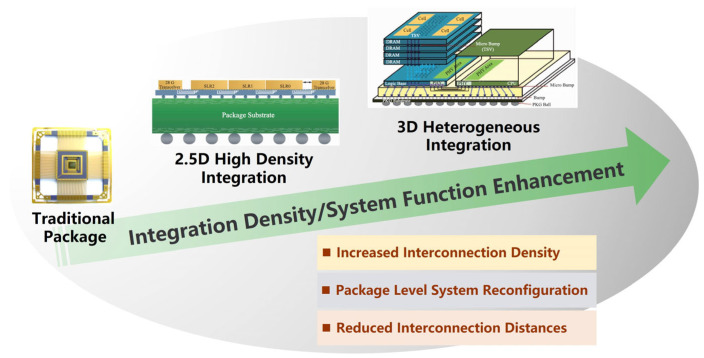
Advanced packaging technical route and characteristics.

As the level of technology continues to increase, the feature size of transistors is approaching the physical limit and it is becoming increasingly difficult to follow Moore’s law [1,2]. Although integrated circuit manufacturers have adopted new technology development and other means, they still cannot meet the current demand for high-performance, miniaturization, intelligence, and low power consumption in electronic devices. The emergence of microsystems provides an important pathway from monolithic integrated planar structure to multi-chip integrated three-dimensional structure. The development of advanced packaging technology provides a technical guarantee for microsystems, and the technical route has evolved from three-dimensional system-level packaging (3D-SiP) to three-dimensional wafer-level packaging (3D-WLP), which has reached the three-dimensional integrated circuit stage (3D-IC) [3]. Advanced packaging, compared with traditional packaging, has characteristics of increased interconnect density, reduced interconnect distance and package level system reconfiguration, etc. [4]. The advanced packaging technical route and characteristics are shown in Figure 1.

**Figure 2:**
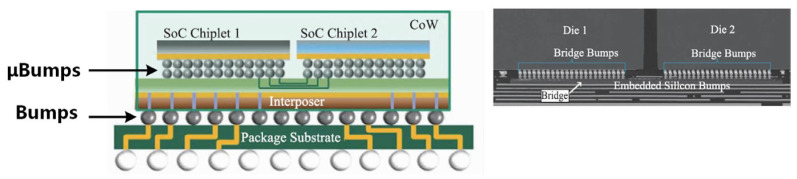
Schematic and structural diagrams of bumps in CoWoS.

The micro bump has become a key technology in advanced packaging structures. Due to its fine pitch and micro-size characteristics, it is widely used in the packaging and integration of CMOS image sensors, HB LED modules, stacked memories, Logic + MemorySiP, Logic and Analog 3D SOC/Sip, etc. [5,6,7,8]. Taking a typical 2.5 D advanced package as an example, CoWoS (chip on wafer on substrate) is a packaging structure based on a silicon adapter board and TSV technology and launched by TSMC in 2012 [9,10]. As shown in Figure 2, logic chips with the same or different functions are interconnected at ultra-short intervals through micro bumps to reduce heat generation and parasitic resistors and capacitors, to achieve the goal of small size, low power consumption, and fewer pins.

Micro bumps play an important role in mechanical support, electrical connection, signal transmission, and heat dissipation in advanced packaging, and their reliability determines the quality of chip packages and even electronic products. Surface morphological defect is one of the main factors affecting the reliability of micro bumps, which are closely related to materials and bonding process parameters. The bump formation process is influenced by the coupling effect of multiple physical fields such as heat, force, and electricity. With the development of micro bump bonding towards high-density and ultra-fine spacing, the microscale effect becomes more apparent [11,12]. With the new features such as lead-free materials and Low K materials, the thermal stress mismatch during the bonding process becomes more severe, which further affects the surface quality of the bumps. The performance of bumps is determined mainly by the material, and selecting the appropriate material is particularly important for improving the reliability and quality of the bumps. Generally, the surface morphology of the micro bump should be flat and consistent. Surface morphology defects of micro bumps refer to rough grain morphology at the interface of micro bumps, resulting in significant IMC coarsening and poor shear performance of micro bumps. Due to periodic changes in temperature during the service of electronic products, the upper and lower surfaces of micro bumps will experience relative motion. As the shear performance of the micro bumps is poor, it will lead to a poor ability to withstand the thermal mismatch between the chip and the substrate, resulting in a decrease in thermal fatigue reliability.

According to the material, bumps can be divided into solder bumps and non-solder bumps. Solder bumps are mainly made of tin-based materials, including SnPb solder and lead-free solders such as SnCu, SnAg, and SnZn; non-solder bumps mainly include Au bumps, Cu bumps, and In bumps [13,14]. The main methods of micro bump preparation include electrodeposition, screen printing, solder injection and evaporation [15,16]. The electrodeposition method has been widely used in practical production because of its simple process, low cost, easy mass production, and high adaptability [17,18].

The effects of different process parameters on electrodeposition were investigated experimentally by Xiaobo Liang et al., Ren et al., and Lee et al. [19,20,21]. Due to limits in experimental techniques, it is not feasible to continuously monitor and capture all the information of the plating growth process dynamically [22,23,24], so experimental methods cannot fully reveal the microscopic process of electrodeposition. However, molecular simulation methods on the atomic scale can help us study the metal crystal growth process from a microscopic perspective. Liu et al. and Treeratanaphitak et al. have simulated the electrodeposition process of Cu and Ag using computer models [25,26]. In this paper, based on molecular simulation theory, the electrodeposition process of Au bumps is simulated by the Kinetic Monte Carlo (KMC) method. The bumps’ deposition growth process and the morphological structure changes under different process parameters are simulated and analyzed, which can optimize the process parameters and enhance the quality of micro bumps.

## 2. Simulation Method and Model Establishment

### 2.1. Simulation Method

The KMC method has become an important for simulating the study of various problems at the atomic scale. It is widely used by researchers in the fields of annealing and recrystallization, metal crystal growth, biochemical reactions, and heat transfer [27,28,29].

To study the influence of electrodeposition parameters on the growth mechanism of gold bump plating layers, the KMC algorithm model is proposed for simulating the electrodeposition growth of gold bumps in this paper. The kinetic processes of deposition adsorption and diffusion migration of deposited atoms on the surface are considered to recast the growth process of bump electrodeposition at the atomic scale in the early stage. Furthermore, the effects of different process parameters on the growth process and surface morphology are analyzed.

The use of Kinetic Monte Carlo to study the atomic deposition process is based on the assumptions that (1) the position of the atoms in each state can be considered as a point on the ideal lattice during the evolution of the model; (2) the state of motion of each atom in the model is only related to its immediate environment and depends on the action of the immediate neighbors on it. Based on these two points, it is possible to build a probabilistic model consistent with the simulated system and to calculate the rate constants for all possible events.

#### 2.1.1. Deposition Event

There are two important parameters in the algorithm, including deposition rate *R_d_* and migration rate *R_h_*. The deposition process refers to the reduction of Au+ into Au atoms, which continuously fall down until reaching a stable atomic layer. The deposition rate represents the number of particles deposited on the substrate surface per unit time. In the KMC simulation of the metal electrodeposition process, its calculation formula is as follows [30]:

(1) $$R_{d}=K_{m}C_{m}e^{\alpha nF_{a}V/RT}$$

where, *K_m_* is the chemical reaction rate constant, *C_m_* is the concentration of Au ion in solution, α is the conversion coefficient of metal atoms in the process of base surface deposition, *n* is the number of ion charges, *F_a_* the Faraday constant, *V* is the deposition voltage, *R* is the ideal gas constant, and *T* is the plating temperature.

#### 2.1.2. Migration Event

Atomic migration means that when Au atoms are deposited in a stable atomic layer, they will also move into the gaps of the layer until it is completely filled with Au atoms. Atomic migration rates are calculated using the Arenius formula:

(2) $$R_{h}=\nu_{0}\exp\left[-\frac{E_{ik}}{K_{B}T}\right]$$

where, *ν*_0_ is the transition frequency, usually taken as 10^12^ s^−1^ or 10^13^ s^−1^ in the KMC simulation; *ν*_0_ is selected as 10^12^ s^−1^ in this model. *E_ik_* represents the energy barrier to be overcome for the first particle to migrate in *k* directions, *T* is the substrate temperature, *K_B_* is the Boltzmann constant.

The energy barrier that needs to be overcome during the migration process of particles is calculated by embedding the embedded atom model (EAM) [31]. The energy of an atomic system can be expressed as:

(3) $$E_{tot}=\sum_{i}E_{i}=\sum_{i}F_{i}\left(\rho_{i}\right)+\frac{1}{2}\sum_{\begin{array}{l} i,j\\ i\neq j \end{array}}\phi \left(r_{ij}\right)$$

(4) $$F\left(\rho_{i}\right)=D\rho_{i}\ln\rho_{i}$$

(5) $$\rho_{i}=\sum_{j}f\left(r_{ij}\right)$$

(6) $$f\left(r_{ij}\right)=A_{2}{\left(r_{c2}-r_{ij}\right)}^{2}\exp(-c_{2}r_{ij})$$

(7) $$\phi \left(r_{ij}\right)=A_{1}{\left(r_{c1}-r_{ij}\right)}^{2}\exp(-c_{1}r_{ij})$$

where *F* is the energy of an atom embedded in an electron cloud of density $\rho_{i}$;$\rho_{i}$ can be viewed as a linear superposition of electron clouds density near the *i*-th atom in the system; *f*(*r_ij_*) is the electron clouds density generated by the atom *j* at the distance *r_ij_*; and *r_ij_* is the distance between atoms *i* and *j*.

The parameters *A*_1_, *A*_2_, *c*_1_, *c*_2_ and *D* required in the EAM model can be obtained by fitting the lattice constant, atomic volume, cohesive energy, single vacancy formation energy, bulk modulus and shear modulus. The EAM model selected in this article was proposed by Doyama et al. [32], and the model parameters are shown in Table 1.

#### 2.1.3. Calculation Method for Deposition and Migration Event Probability

The essence of the KMC method is to randomly select the current occurrence of an event at a certain moment based on the weight of the probability of occurrence of each event [33].

The probability of occurrence of the deposition process *P_d_* is:

(8) $$P_{d}=\frac{R_{d}}{R_{d}+\sum_{i=1}^{M}R_{i}}$$

The probability of occurrence of the migration process *P_h_* is:

(9) $$P_{h}=\frac{\sum_{i=1}^{M}R_{i}}{R_{d}+\sum_{i=1}^{M}R_{i}}$$

After the migration process occurs, the probability of the *i*-th migration event occurring among all migration events is:

(10) $$P_{m}=\frac{R_{m}}{\sum_{i=1}^{M}R_{i}}$$

In the program model, the probabilities of all events in the system are calculated first, and then a random number is generated to determine whether the system undergoes deposition or migration [34,35,36]. If a deposition process occurs, the continued generation of random numbers determines the initial position and falling direction of the deposition atoms; If a migration process occurs, a certain atom in the system is randomly selected to complete the migration process based on the probability of each migration event occurring.

#### 2.1.4. Simulation Algorithm Flow

**Figure 3:**
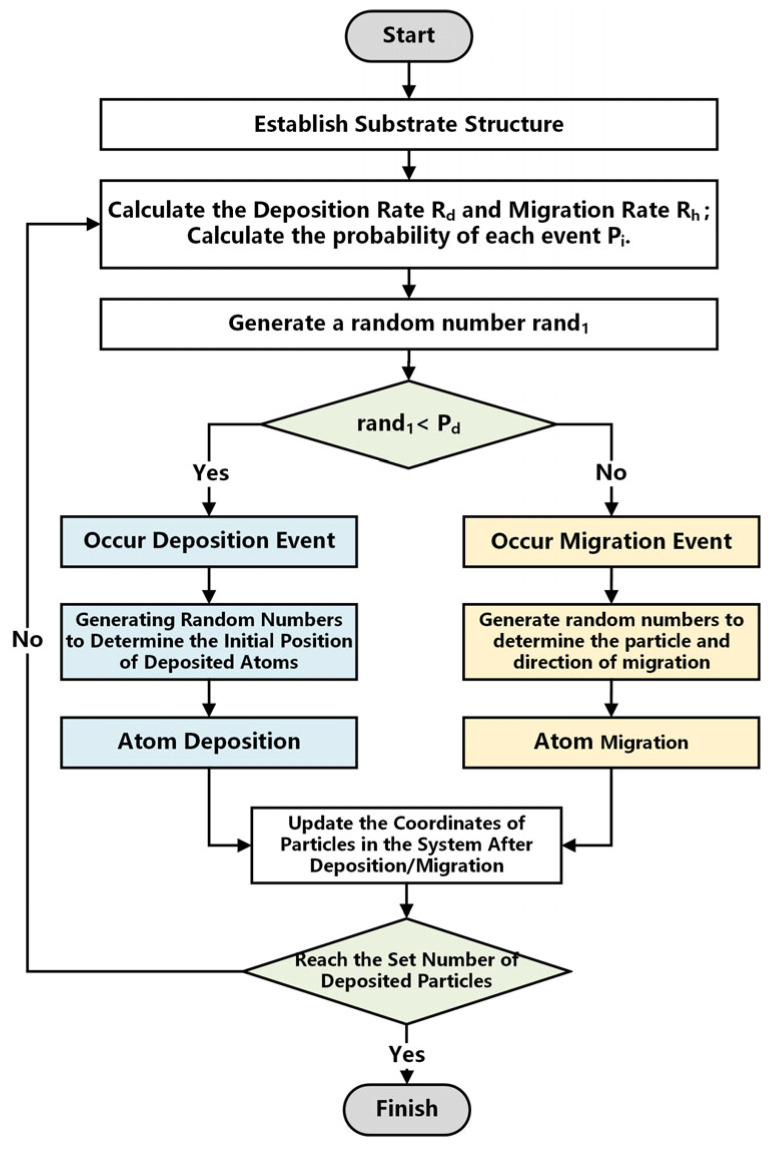
Atom deposition process algorithm flowchart.

The atom deposition process algorithm based on the KMC method is written in C# using the Visual Studio development platform. The algorithm process can be described as follows. The algorithm flowchart is shown in Figure 3.

(1) Initialize the substrate structure and calculate the deposition rate *R_d_* and migration rate *R_h_* arrays.
(2) Calculate the probability of each event *P_i_*.
(3) Generate a random number 0 < rand_1_ < 1 by a random number algorithm.
(4) Compare the deposition event probability *P_d_* with the random number rand_1_, and if rand_1_ < *P_d_*, select the deposition event; otherwise, the migration event is selected.
(5) Execute the selected migration event or deposition event.
(6) Update the coordinate array and event probability array of migrated or deposited particles and particles in the immediate vicinity.
(7) Repeat steps 2–7 until the set number of deposited particles is reached.
(8) Output and count the position coordinates of all particles in the system, calculating the simulation results.

### 2.2. Computation Models

**Figure 4:**
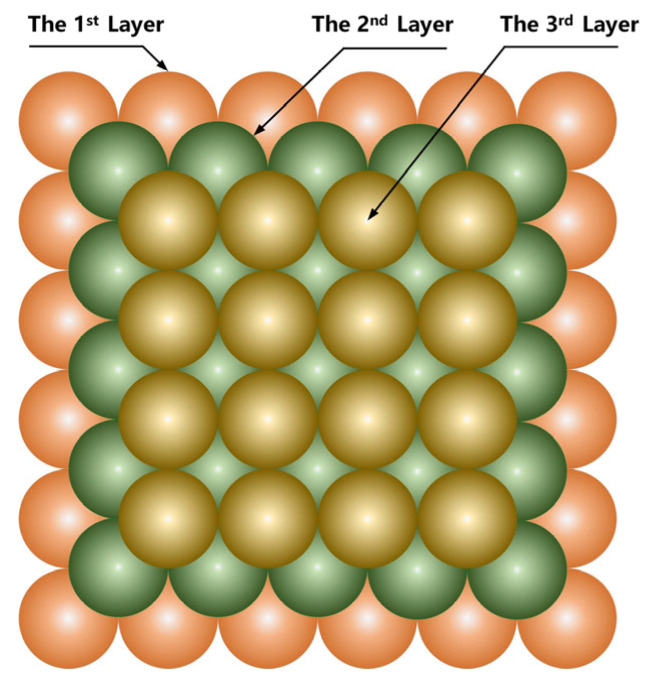
Structural model of deposited atoms.

As the deposition of the bump plating layer mainly occurs on the UBM (Under Bump Metallization) infiltrating layer, the substrate model of the Au infiltrating layer is constructed in the model first. Then, the deposition and growth of the Au plating layer are simulated and studied based on the model above. The physical evolution behavior between particles in the model includes the diffusion migration behavior of Au^+^ in solution due to electrochemical reduction to atoms deposited on the substrate surface and the diffusion migration behavior of deposited Au atoms on the substrate surface. As the dissolution of deposited atoms is difficult, and as it has little effect on the deposition process, it is not considered in the model. The structure of the deposited atoms is shown in Figure 4, with three colored balls representing three different atomic layers. In the face-centered cubic structure, atoms in different layers are staggered. In the simulation, the size of the substrate is set as 14.419 nm × 14.419 nm. A total of 50,000 Au atoms are deposited on the substrate. The values of the parameters in the simulation are shown in Table 2.

**Figure 5:**
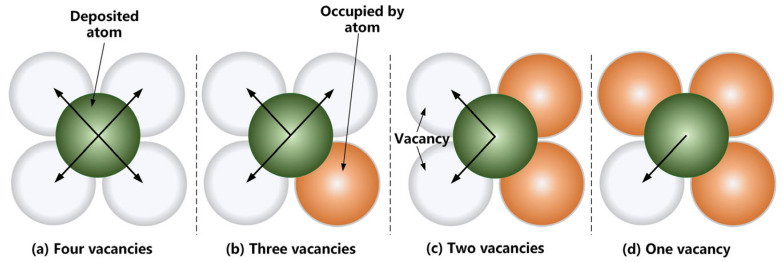
Four situations where vacancies appear in the lower layer of deposited atoms.

The process in which Au^+^ in the plating solution is reduced to atoms and deposited on the substrate surface is called a deposition event [37]. Figure 5 shows the different numbers of vacancies in the lower layer of deposited atoms and the direction in which the deposited atoms may fall. The green atoms indicate the deposited atoms in the upper layer, the white atoms indicate that there are no atoms in the lower layer at this position; and the orange atoms indicate that there are atoms in the lower layer of this position.

## 3. Analysis and Results

In the simulation model, three process factors on the growth and surface morphology of the electroplated layer are considered, namely the deposition voltage, the temperature of the plating solution and the ion concentration. After the deposition of all the atoms is completed, the coordinate information of all the atoms is counted and output. Then, the three-dimensional topography of the coating atoms is obtained, and the influence of different electrodeposition parameters on the topography of the coating is analyzed.

In the simulation, the surface roughness is used to characterize the surface quality of the deposited film. After the deposition is completed, the coordinate height values of all the surface atoms of the film are extracted, and then the surface roughness is calculated by two measurement methods in precision metrology. The calculation formula are as follows [38,39]:

(11) $$R_{a}=\frac{\sum_{i=1}^{N}\left|Z_{i}^{}-\bar{Z}\right|}{N}$$

(12) $$R_{q}=\sqrt{\frac{\sum_{i=1}^{N}{\left(Z_{i}^{}-\bar{Z}\right)}^{2}}{N}}$$

where, $R_{a}$ is the average roughness, $R_{q}$ is the root mean square roughness, $Z_{i}$ is the coordinate height of the *i*_th_ atom on the film surface, $\bar{Z}$ is the average of the coordinate heights of all surface atoms, and *N* is the number of all surface atoms.

### 3.1. The Effect of a Single Factor on Surface Roughness

#### 3.1.1. Effect of Deposition Voltage on Surface Morphology

**Figure 6:**
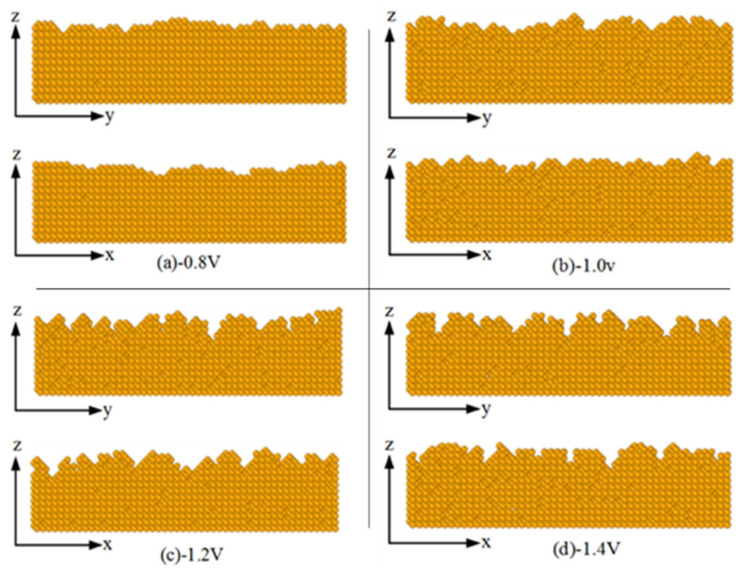
The influence of deposition voltage on cross-section morphology.

In the simulation, the ion concentration *C*_m_ is set to 0.05 mol/L, the plating temperature (T) is 328 K, and the deposition voltage (V) ranges from −0.8 V to −1.4 V. Figure 6 is a cross-sectional view of the coating atoms along with the x-axis and y-axis directions after the simulation. It can be found that when the deposition voltage is −0.8 V, the atom height distribution on the surface of the deposition layer is relatively uniform, and the surface structure is flat and smooth. As the voltage increases to −1.0 V, small bumps start to appear on the surface of the deposited layer. When the voltage continues to increase to −1.2 V and −1.4 V, the difference in the height of surface atoms increases. The phenomenon of an obvious island-like structure appears, with a large number of vacancies and defects.

**Figure 7:**
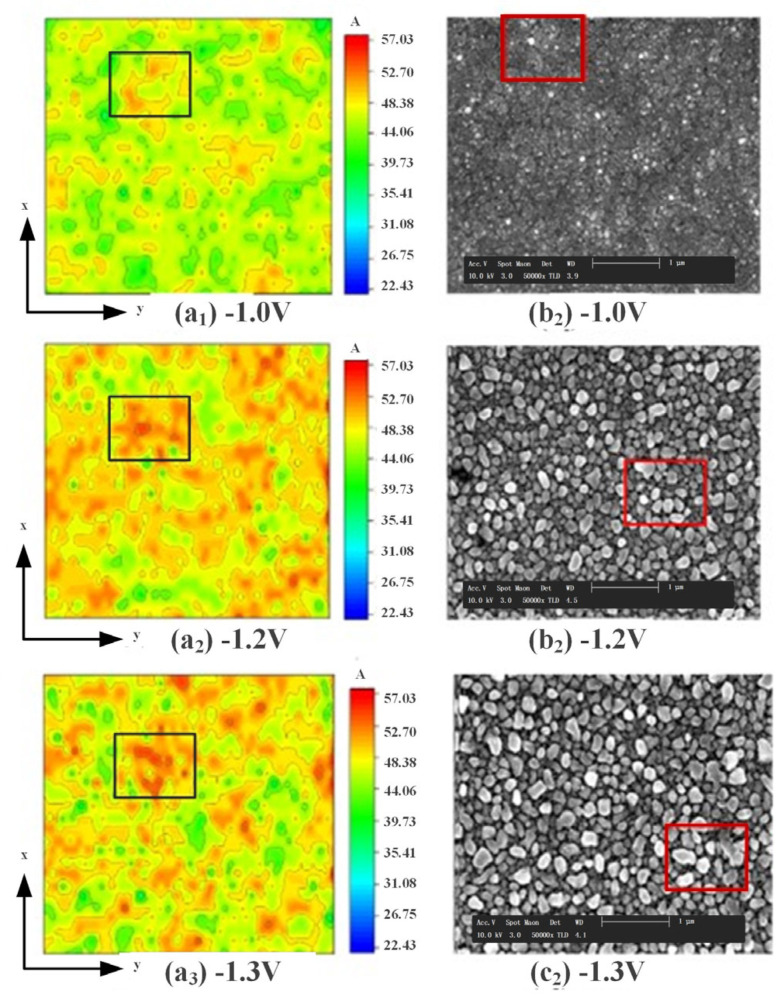
The comparison of simulation and experimental under different deposition voltages.

Figure 7 shows the results of the simulations in this paper compared with those obtained by Ghezali et al. [40] in their experiments on electrodeposited ZnS plating. The Figure 7a_1_–a_3_ show the simulation results of the surface atoms of the coating under different deposition voltages in the KMC simulation in this paper; the Figure 7b_1_–b_3_ show the scanning electron microscope (SEM) scanning electron surface topography obtained by Ghezali et al. when preparing ZnS coatings under different voltages. It can be seen from the figure that as the voltage increases from −1.0 V to −1.3 V, the proportion of isolated islands and vacancies on the surface of the ZnS coating increases significantly, and the uniformity of the atom height distribution becomes even worse.

#### 3.1.2. Effect of Plating Temperature on Surface Morphology

**Figure 8:**
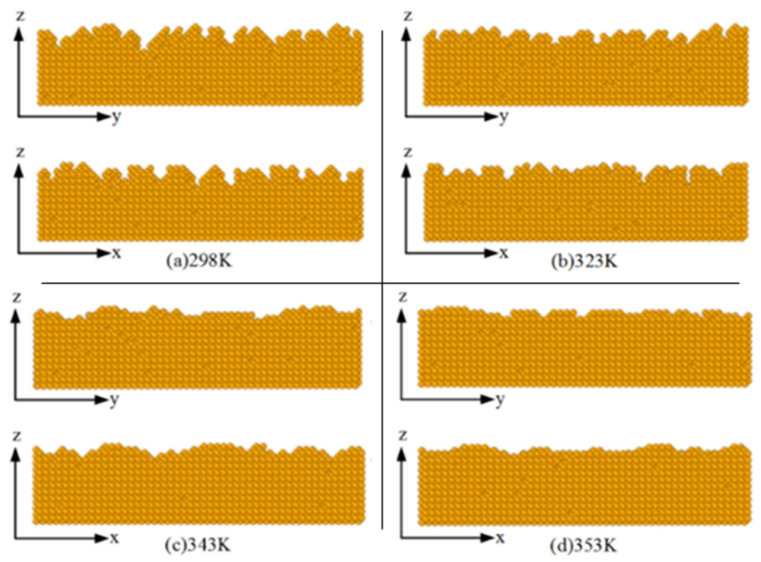
The influence of plating temperature on cross-section morphology.

In the simulation, the concentration *C*_m_ is 0.075 mo1/L, the deposition voltage (V) is 1.0 V, and the variation of the plating temperature (T) is from 298 K to 353 K. The cross-sectional shape of the plating layer after deposition is shown in Figure 8. When the temperature is 298 K, the height distribution of the surface deposition atom is very uneven. A large number of bumps and pits appear, producing a large number of “gullies”. As the temperature rises to 323 K, the surface gradually becomes flat, but there is still a certain number of vacancies and defects. As the temperature continues to increase to 343 K and 353 K, the smoothest cross-sections are deposited, with significantly reduced roughness and highly uniform distribution of surface atoms.

**Figure 9:**
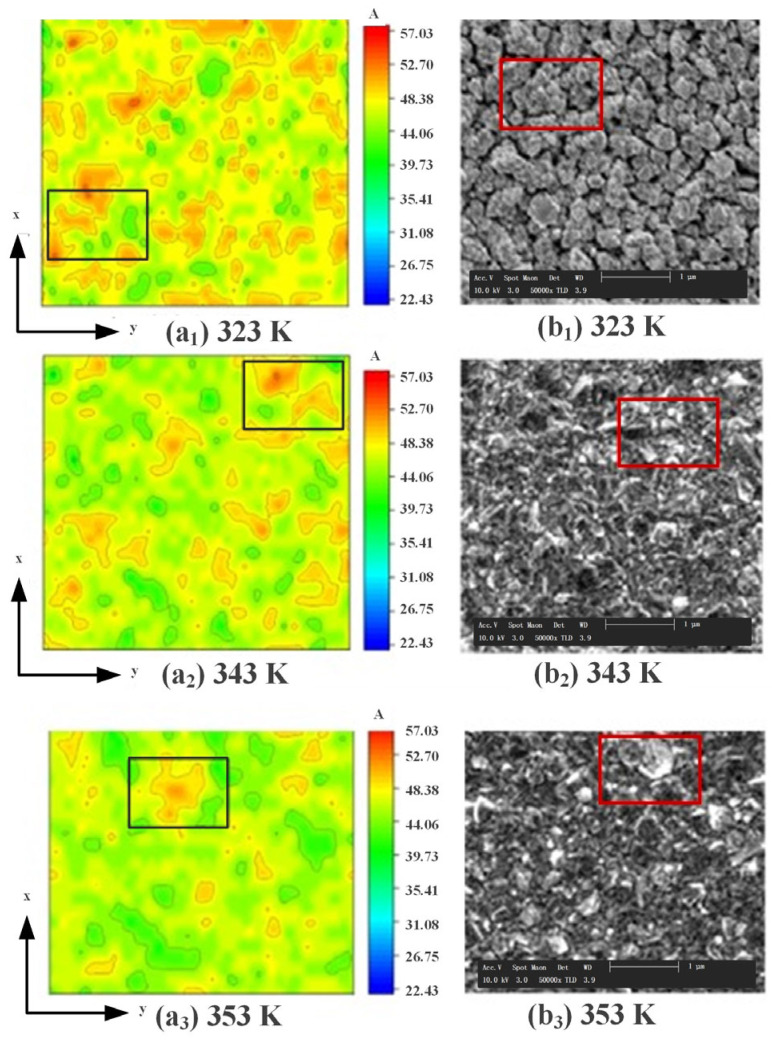
The comparison of simulation and experimental under different plating temperatures.

Figure 9 shows the results of the simulation surface morphology by the KMC method in this paper compared with those microscopic morphology of the Au layers preparing by Pan Jianling et al. [41] in their Au plating experiments at different plating temperatures. Figure 9a_1_–a_3_ show the surface morphologies of the coating obtained at different plating temperatures in the KMC simulation in this paper; Figure 9b_1_–b_3_ show the SEM scanning electron surface topography obtained Pan Jianling et al. when preparing Au coatings under different voltages. It can be observed that when the electroplating temperature is 323 K, there are more pores between the grains of the Au coating surface, and the surface morphology is rough, as shown in Figure 9a_1_,b_1_; as the plating temperature increases to 343 K, the coating structure becomes dense and flat, and the roughness decreases as shown in Figure 9a_2_,b_2_; when the temperature reaches 353 K, the obtained coating surface is the smoothest and the flattest, and the surface quality is the best, as shown in Figure 9a_3_,b_3_. It can be seen that the experimental results obtained by Pan Jianling et al. are consistent with the simulation results of this paper.

#### 3.1.3. Effect of Ion Concentration on Surface Morphology

**Figure 10:**
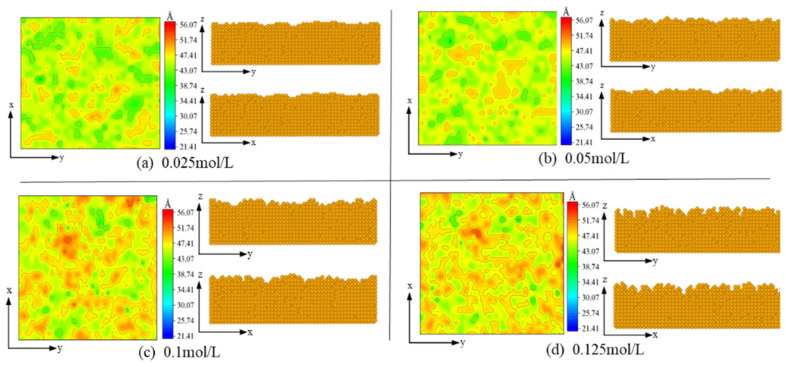
The influence of ion concentration on cross-section morphology.

Based on the simulation model, the deposition voltage (*V*) is set as −0.8 V and the temperature of the plating solution (*T*) is set as 328 K. The Au^+^ concentration in the plating solution is varied to study the effect of different ion concentrations on the electrodeposition process. Figure 10 shows the cross-sectional view of the coating structure and the image of the surface atom height obtained after the simulation at different concentrations. The results show that at concentrations of 0.025 mol/L and 0.05 mol/L, the atoms on the surface of the films are uniformly distributed, the number of vacancies and isolated islands is less, and the surface quality is good, as shown in Figure 10a,b. As the concentration increases to 0.1 mol/L, the bumps and pits on the surface of the plating are increased and the surface atomic distribution starts to become uneven, as shown in Figure 10c. When the concentration reaches 0.125 mol/L, the roughness of the plating surface increases significantly, producing more “gullies”, and the number of vacant sites increases significantly, showing island-like pattern growth, as shown in Figure 10d. This is mainly because the electrodeposition rate is influenced by the concentration factor, where an increase in concentration increases the deposition rate of atoms, and atoms deposited onto the electrode surface are easily buried by the newly deposited atoms and cannot migrate. It can be seen that the ion concentration does not affect the surface morphology to the same extent as the deposition voltage and the temperature of the plating solution.

### 3.2. The Effect of Multiple Parameters on Surface Roughness

The root mean square (RMS) roughness *R*_q_ of the plated surface is calculated by equation after the simulation. In this paper, the effect of two-parameter coupling on surface roughness is analyzed. In the KMC simulation, the deposition voltage varied from −0.8 V to −1.4 V, the ion concentration varied from 0.025 mol/L to 0.125 mol/L, and the plating solution temperature varied from 298 K to 358 K.

#### 3.2.1. Effect of Deposition Voltage and Ion Concentration on Roughness

**Figure 11:**
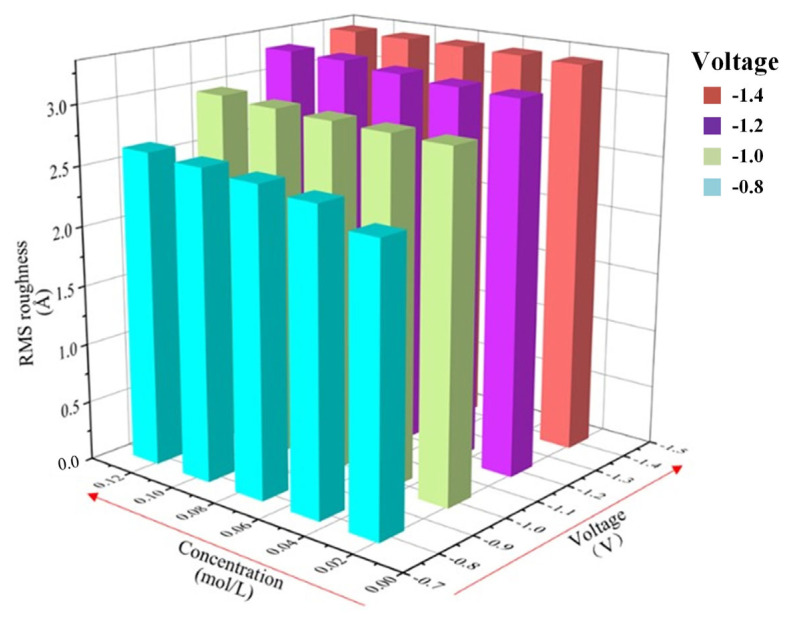
The RMS roughness histogram for coupled parameters of deposition voltage and ion concentration.

The plating temperature is constant at 328 K while the values of the deposition voltage and ion concentration vary to calculate and analyze the RMS roughness of the coating surface. The data are shown in Table 3, and the variation of roughness with voltage versus concentration is plotted in Figure 11.

Overall, the coatings have better surface quality at lower ion concentrations and lower deposition voltages. The effect of changing deposition voltages on roughness is more pronounced at lower concentrations. Under the same ion concentration, as the deposition voltage increases from −0.8 V to −1.4 V, when the ion concentration is 0.025 mol/L, the surface roughness increases by 38.8%. As the deposition voltage increases from −0.8 V to −1.4 V, the surface roughness increases by 25.5%. Under the same deposition voltage, when the deposition voltage is −1.4 V, as the ion concentration decreases from 0.125 mol/L to 0.025 mol/L, the surface roughness decreases by 0.75%. When the deposition voltage is −0.8 V, the surface roughness decreases by 2.6%. In general, when the deposition voltage is −0.8 V and the ion concentration is 0.025 mol/L, the surface quality of the coating is the best, and the RMS roughness is the smallest. The minimum value is 2.3631 Å.

#### 3.2.2. Effect of Deposition Voltage and Plating Temperature on Roughness

**Figure 12:**
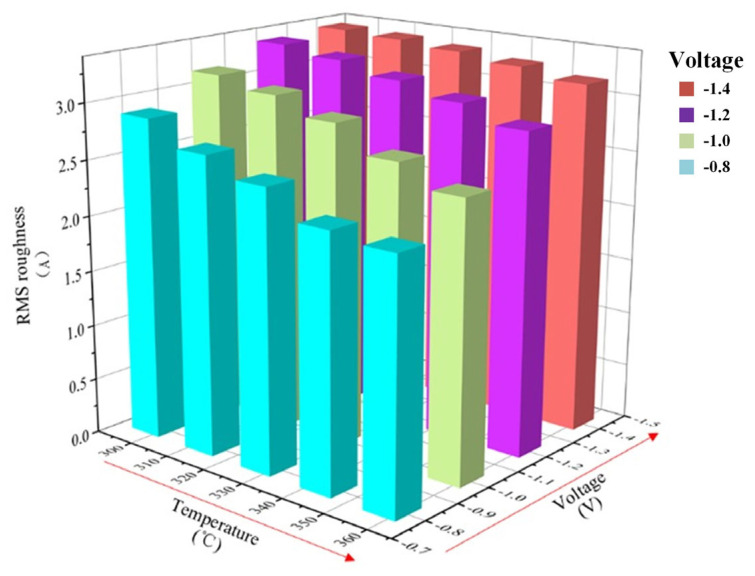
The RMS roughness histogram for coupled parameters of deposition voltage and plating solution temperature.

The ion concentration constant at 0.05 mol/L in the model, and the values of plating solution temperature and deposition voltage are set to vary to calculate and analyze the RMS roughness of the coating surface. The data are shown in Table 4 and the variation of roughness with voltage and temperature is plotted in Figure 12.

Overall, the coating has better surface quality at lower deposition voltage and higher temperature, and the effect of changing deposition voltage on surface roughness is more pronounced at higher temperatures. When the deposition voltage is the same, the surface roughness decreases with the increasing of temperature. When the deposition voltage is −0.8 V, the temperature increases from 298 K to 358 K, and the surface roughness decreases by 23.2%. When the deposition voltage is −1.4 V, the temperature increases from 298 K to 358 K, and the surface roughness decreases by 5.7%. When the temperature is the same, the surface roughness decreases with the increasing of deposition voltage. When the temperature is 298 K, the deposition voltage increases from −0.8 V to −1.4 V, and the surface roughness increases by 15.9%; when the temperature is 358 K, the deposition voltage increases from −0.8 V to −1.4 V, and the surface roughness increases by 42.4%. On the whole, when the deposition voltage is −0.8 V and the plating temperature is 358 K, the surface quality of the coating is the best, and the RMS roughness is the smallest. The minimum RMS roughness is 2.2179 Å.

#### 3.2.3. Effect of Plating Solution Temperature and Ion Concentration on Roughness

**Figure 13:**
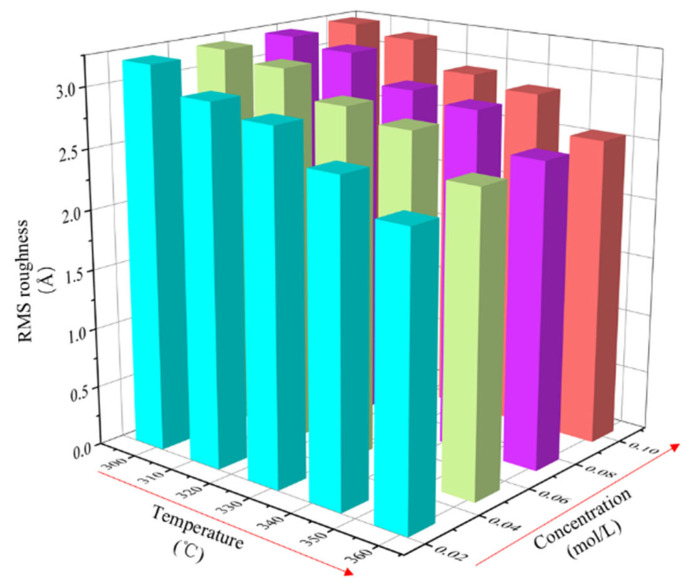
The RMS roughness histogram for coupled parameters of plating solution temperature and ion concentration.

In the model, the value of the deposition voltage (V) is kept unchanged at −1.0 V. The values of the plating temperature and ion concentration are set as variable to calculate and analyze the RMS roughness of the coating surface. The data are shown in Table 5. The variation of roughness with plating solution temperature and ion concentration is plotted in Figure 13.

## 4. Conclusions

In this paper, the process of preparing gold bumps by electrodeposition was simulated by the Kinetic Monte Carlo method. From the microscale perspective, the mechanism of the bump growth process was investigated, and the effects of different deposition parameters on the layer growth process were studied. Furthermore, the differences in the surface morphology and roughness of the layers under different deposition parameters were compared and analyzed. The main conclusions are as follows:

(1) From the perspective of the single-factor, lower deposition voltage, lower ion concentration and higher plating solution temperature can help reduce the number of empty spaces and isolated islands on the surface of the electrodeposited layer, resulting in a flat and smooth surface topography.
(2) From the perspective of the multi-factor, the simulation show that the deposition voltage and deposition temperature have a greater effect on the surface RMS roughness and play a dominant role, while the ion concentration has a smaller effect on the RMS roughness.

## Figures and Tables

**Table 1:** The Parameter Values of Au Metal in EAM model.

Model Parameter	Values
*A*_1_	518,021.8789
*A*_2_	0.0183
*c*_1_	15.5770
*c*_2_	1.2867
*D*	11.9318

**Table 2:** The parameters and values of electrodeposition simulation.

Number	Symbol	Set Value	Description
1	*N_dep_*	50,000	Number of deposited Au atoms
2	*a*	14.419 nm	Length of the base in the X direction
3	*b*	14.419 nm	Length of the base in the Y direction
4	*K_m_*	0.2 × 10^−3^ m^3^/mol·s	Chemical reaction rate constant
5	*F_a_*	96,485.338 C/mol	Faraday constant
6	*R*	8.314 J/mol·K	Ideal gas constants
7	α	0.5	Conversion coefficient
8	*n*	1	Ionic charge number
9	*T*	298 K~358 K	Plating temperature
10	*V*	−0.8 V~−1.4 V	Deposition voltage
11	*C_m_*	0.025 mol/L~0.125 mol/L	Ion concentration

**Table 3:** RMS roughness values for coupled parameters of deposition voltage and ion concentration.

	Voltage (V)	−0.8	−1.0	−1.2	−1.4
Concentration (mol/L)					
0.025		2.3631	2.8953	3.1283	3.2813
0.05		2.5166	2.9084	3.1444	3.2925
0.075		2.5699	2.9219	3.1798	3.2998
0.1		2.6039	2.9430	3.2230	3.3030
0.125		2.6364	2.9743	3.2361	3.3078

**Table 4:** RMS roughness values for coupled parameters of deposition voltage and plating solution temperature.

	Voltage (V)	−0.8	−1.0	−1.2	−1.4
Temperature (K)					
298		2.8917	3.1483	3.3173	3.3532
313		2.6769	3.0547	3.2481	3.3309
328		2.5166	2.9064	3.1454	3.2925
343		2.2763	2.6752	3.0363	3.2347
358		2.2179	2.4903	2.8901	3.1591

**Table 5:** RMS roughness values for coupled parameters of plating solution temperature and ion concentration.

	Concentration (mol/L)	0.025	0.05	0.075	0.1
Temperature (K)					
298		3.1975	3.2143	3.2232	3.2359
313		2.9890	3.1358	3.1577	3.1673
328		2.8953	2.9164	2.9256	2.9430
343		2.6262	2.8182	2.8507	2.8583
358		2.3571	2.4909	2.5421	2.5623

## Data Availability

The data presented in this study are available on request from the corresponding author. The data are not publicly available due to a confidentiality request.
